# Line-Field Confocal Optical Coherence Tomography for the Evaluation of Pigmented Skin Lesions of the Genital Area

**DOI:** 10.3390/diagnostics15233023

**Published:** 2025-11-27

**Authors:** Simone Cappilli, Gerardo Palmisano, Elisa Cinotti, Lucas Boussingault, Luca Pellegrino, Linda Tognetti, Simona Maria Fragomeni, Javiera Pérez-Anker, Jean-Luc Perrot, Angela Santoro, Giorgia Garganese, Gian Franco Zannoni, Mariano Suppa, Ketty Peris, Alessandro Di Stefani

**Affiliations:** 1 Dermatologia, Dipartimento di Medicina e Chirurgia Traslazionale, Università Cattolica del Sacro Cuore, 00168 Rome, Italy; simo.cappilli@gmail.com (S.C.);; 2 UOC di Dermatologia, Dipartimento di Scienze Mediche e Chirurgiche, Fondazione Policlinico Universitario A. Gemelli IRCCS, 00168 Rome, Italy; alessandro.distefani@policlinicogemelli.it; 3 Dermatology Unit, Department of Medical, Surgical and Neurosciences, University of Siena, 53100 Siena, Italy; 4 Department of Dermatology, Hôpital Erasme, Université Libre de Bruxelles, 1070 Brussels, Belgium; 5 Department of Dermato-Oncology, Institute Jules Bordet, Université Libre de Bruxelles, 1070 Brussels, Belgium; 6 UOC Ginecologia Oncologica, Dipartimento per le Scienze della Salute della Donna e del Bambino e di Sanità Pubblica, Fondazione Policlinico Universitario A. Gemelli IRCCS, 00168 Rome, Italy; 7 Melanoma Unit, Hospital Clinic Barcelona, University of Barcelona, 08007 Barcelona, Spain; 8 CIBER de Enfermedades Raras, Instituto de Salud Carlos III, 08034 Barcelona, Spain; 9 Department of Dermatology, University Hospital of Saint-Etienne, 42270 Saint-Etienne, France; j.luc.perrot@chu-st-etienne.fr; 10 Pathology Unit, Department of Woman and Child’s Health and Public Health Sciences, Fondazione Policlinico Universitario A. Gemelli IRCCS, 00168 Rome, Italy; angela.santoro@policlinicogemelli.it (A.S.);; 11 Pathology Institute, Catholic University of Sacred Heart, 00168 Rome, Italy; 12 Unità Operativa di Chirurgia degli Organi Genitali Esterni Femminili, Divisione di Ginecologia Oncologica, Dipartimento per le Scienze della Salute della Donna, del Bambino e di Sanità Pubblica, Fondazione Policlinico Universitario A. Gemelli IRCCS, 00168 Rome, Italy; 13 Gemelli Women’s Health Center for Digital and Personalized Medicine, Dipartimento Universitario Scienze della Vita e Sanità Pubblica, Sezione di Ginecologia ed Ostetricia, Università Cattolica del Sacro Cuore, 00168 Rome, Italy; 14 Groupe d’Imagerie Cutanée Non Invasive (GICNI) of the Société Française de Dermatologie (SFD), 75008 Paris, France

**Keywords:** diagnostic imaging, LC-OCT, genital skin lesions, skin tumors, clinical dermatology

## Abstract

**Background/Objectives:** Pigmented lesions of the genital area are of commonly occurrence, with population-based incidence estimated around 10–20%. Historically, invasive biopsy or surgical removal were recommended to obtain a definite diagnosis. Line-field confocal optical coherence tomography (LC-OCT) is a novel multimodality imaging tool, able to reproduce a “virtual biopsy” of skin lesions, offering vertical, horizontal, and three-dimensional (3D) imaging down to the mid-dermis with high-resolution real-time visualization. The aims of the study were (i) to describe the LC-OCT features in a series of benign and malignant pigmented genital lesions (PGLs), (ii) to investigate the impact of LC-OCT on the diagnostic accuracy, (iii) and to estimate the diagnostic concordance between LC-OCT and histopathology. **Methods:** This was a retrospective, cross-sectional study including histologically confirmed PGL investigated with LC-OCT over 2 years. Descriptive statistics were calculated for continuous and categorical variables. Diagnostic accuracy of LC-OCT and dermoscopy was compared, and the LC-OCT–histopathology diagnostic concordance was estimated. **Results:** A total of 96 PGLs were analyzed in 23 male and 57 female patients. Pathologic reporting consisted of 56 melanoses, 21 nevi (15 compound nevi and 6 atypical genital nevi), 10 melanomas, and 9 angiokeratomas. LC-OCT obtained higher diagnostic accuracy rather than dermoscopy; diagnostic concordance LC-OCT/histopathology was 91% (87/96; 95% CI 83.7–95.3). Melanoses were characterized by a continuous undulated junction. Common genital nevi were identified by the presence of regular dense nests, while dischoesive nests were seen in atypical genital nevi. Features of epidermal disarray with pagetoid cells, junctional atypia, and discohesive nests were observed in melanoma. Angiokeratomas showed the presence of dark vascular lacunae. **Conclusions:** Providing in vivo key clues with a resolution close to classic histopathology, LC-OCT may have a valuable role in the clinical management of PGLs, particularly when lesions involve large areas or are multiple in number.

## 1. Introduction

Pigmented lesions of the genital area are a common occurrence, with population-based incidence estimated of 10–20% [1,2]. Pigmentation is mainly due to the development of melanotic macules (also called melanoses), less frequently of melanocytic (naevi and melanoma), non-melanocytic (epithelial skin cancers, seborrheic keratoses, and post-inflammatory pigmentations), and vascular skin lesions (angiokeratomas). They most commonly arise on the labia majora in women and the sheath in men [3].

A correct diagnosis of pigmented genital lesions (PGLs) is of paramount importance for proper management, also considering that this area may go unnoticed for a long period due to its location [1,2,3,4]. Historically, invasive biopsy or surgical removal of all PGLs was recommended by a dermatologist and gynecologist to obtain a definite diagnosis [4,5]. In recent decades, the growing application of advanced skin imaging techniques, such as dermoscopy and reflectance confocal microscopy (RCM), has made it possible to consider a more conservative approach for benign PGL, moving forward from the old clinical approach. This paradigm shift was the result of increased diagnostic accuracy obtained by the integration of in vivo tools, dermoscopy, and RCM, in association to clinical evaluation [6,7,8,9,10]. As mucosal melanoma is 2.25 times more lethal than cutaneous melanoma, its early detection provides the best chance of survival [11].

Line-field confocal optical coherence tomography (LC-OCT) is a novel multimodal imaging tool able to reproduce a “virtual biopsy”, and offering a real-time visualization of skin lesions in vertical and horizontal view and 3D mode. Merging the technical skills of optical coherence tomography (OCT) and RCM, it overcomes the limitations of these techniques in terms of the depth penetration of the image, its spatial resolution, and image orientation. Advanced algorithms (often using machine learning or deep learning) automatically segment and define tissue layers (e.g., epidermis, dermal–epidermal junction, and dermis) for 3D reconstruction [12,13,14,15]. Upgrowing data from the literature support its beneficial role for the diagnosis of skin tumors and inflammatory and infectious diseases [16,17,18]. Also, tumor margins have been pre-operatively assessed through LC-OCT [16]. Recently, in a case series including nine patients, distinct LC-OCT findings were observed in benign and malignant PGLs, suggesting that this device may play an additional role in the in vivo evaluation of this sensitive area [19].

The aims of this study were (i) to describe the LC-OCT features of benign and malignant PGLs in a cohort of patients, (ii) to investigate the impact of LC-OCT on diagnostic accuracy, (iii) and to estimate diagnostic concordance between LC-OCT and histopathology.

## 2. Materials and Methods

Sample population. This was a retrospective, monocentric, cross-sectional study evaluating histologically confirmed PGLs. Inclusion criteria were the availability of clinical, dermoscopic, and LC-OCT images of each PGL over 2 years (January 2022–January 2024), as well as patient demographics (age, sex, skin phototype, and number of lesions).

Ethics committee approval was waived because the study affected neither the routine diagnostic nor therapeutic management of these patients.

LC-OCT technical properties, imaging acquisition, and evaluation. The CE-marked DeepLive LC-OCT device (DAMAE Medical^®^, Paris, France) was used for the imaging acquisitions. This technique can acquire high-resolution images/videos in vertical and horizontal mode (≅1.3-µm lateral resolution, ≅1.1-µm axial resolution), with a maximum depth reaching the superficial/mid-dermis (≅500 µm). It may also generate 3D cubes/slices in areas of major interest. The integration of a dermoscopic camera within the handheld probe allows the simultaneous viewing of LC-OCT/dermoscopic images on the screen, working as location tracking during the examination. At least one high-quality video (vertical and horizontal) and three vertical and horizontal images were considered for the external evaluation. Horizontal images were chosen at different skin levels (epidermis, dermal–epidermal junction, and superficial dermis). Three-dimensional reconstruction, if available, was included in the image analysis.

Dermoscopic and LC-OCT images/videos were evaluated by 2 independent experts in noninvasive skin imaging (E.C. and L.T.) who suggested the most likely diagnosis based on an overall evaluation of the criteria for each lesion (“holistic approach”). A pathological report was considered the gold standard for the definite diagnosis.

LC-OCT diagnostic algorithm. For each lesion, vertical and horizontal LC-OCT sections and, when available, 3D reconstructions were reviewed. Classification was based on predefined morphological criteria derived from the reflectance confocal microscopy literature and preliminary LC-OCT observations [8,9,11,12,16,17,19].

During image review, readers assessed epidermal architecture, the appearance and continuity of the dermoepidermal junction (DEJ), the presence and distribution of melanocytic nests, the occurrence of pagetoid bright cells, and the morphology of dermal structures.

Once feature assessment was completed, an overall diagnosis was assigned by integrating all morphological criteria according. In case of discordance between readers, a consensus diagnosis was established through joint review. Histopathology served as the reference standard for all diagnostic categories.

Statistical analysis. LC-OCT horizontal-section images were investigated based on already established RCM criteria for PGLs, while vertical images were evaluated according to preliminary LC-OCT data on pigmented lesions [7,8,13]. Additional LC-OCT criteria were introduced if required.

The analysis comprised descriptive statistics, as median and minimum–maximum values were calculated for continuous variables, and frequencies (*n*) and percentages (%) for categorical variables. Diagnostic accuracy was estimated comparing dermoscopy and LC-OCT, and the diagnostic concordance comparing LC-OCT to histopathology. The Fisher exact test was used for the comparison of different LC-OCT parameters between malignant and benign lesions, and their association with histological diagnosis. A *p* value < 0.05 was considered statistically significant. All analyses were performed using R statistical software (version 4.4.1; R Foundation for Statistical Computing, Vienna, Austria).

## 3. Results

Demographic data of the samples are listed in Table 1. A total of 96 PGLs were investigated in 23 male and 57 females (mean age of 46.1 years old). In total, 12 patients presented with multiple lesions, all of which were diagnosed as melanoses, while the remaining 68 patients had a single lesion. Pathologic report consisted of 56 melanoses, 21 nevi [(15 compound nevi and 6 atypical genital nevi (AGN)], 10 melanomas, and 9 angiokeratomas (AKs). Melanoses were mainly localized on labia minora in women (21/37, 45%), and on the penis in men (13/19, 45%); nevi mostly occurred on labia majora in women (4/9, 45%) and on the penis in men (5/12, 41%); angiokeratomas mostly occurred on the pubis in women (2/9, 22%) and on the scrotum in men (6/9, 67%). All melanomas (10/10, 100%) found in women were on the labia minora and pubis (Table 1).

LC-OCT features of the benign and malignant PGLs are described below in dual-mode (vertical and horizontal) for each entity. The frequency of all parameters is collected in Table 2 (vertical criteria) and Table 3 (horizontal criteria).

### 3.1. LC-OCT Vertical Features of Benign PGLs

**Figure 1:**
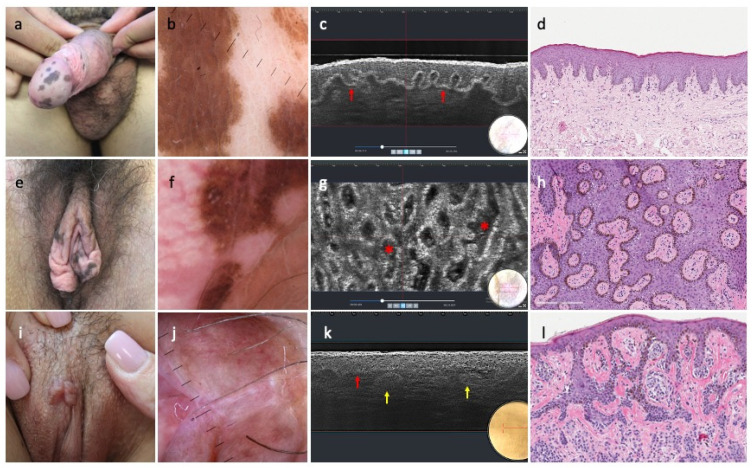
Clinical, dermoscopy, mono-dimensional LC-OCT, and histopathology of melanosis and congenital nevus: (**a**) multiple melanosis on the glans, foreskin, and penis; (**b**) revealing parallel pattern on dermoscopy; (**c**) vertical LC-OCT showing undulated continuous bright junction (red arrows); (**d**) matching with elongated rete ridges with hyperpigmentation of the basal layer (HE, original magnification × 150); (**e**) multiple melanosis on the labia majora and minora; (**f**) showing a mixed dermatoscopic pattern; (**g**) horizontal LC-OCT unveiling a draped pattern (red asterisks); (**h**) exactly corresponding to horizontal histologic exam, showing hyperpigmentation of the basal keratinocytes and a slight increase in melanocytes (HE, original magnification × 150); (**i**) congenital nevus on the labia minora; (**j**) with structureless areas and vessels on dermoscopy; (**k**) vertical LC-OCT displaying a well-defined bright junction (red arrow) and regular nests (yellow arrows) in the upper dermis; (**l**) as observed at the corresponding histologic with elongated rete ridges and melanocytic nests (HE, original magnification × 150).

Melanoses largely showed a regular epidermis (43/56, 77%) and a continuous undulated junction (47/56, 84%). In 12/56 (21%) of lesions we observed atypical cells in the epidermis and at the junction, localized or sparse; 14/56 (25%) of cases showed clusters of plump bright cells at depth in the dermis (Figure 1).

**Figure 2:**
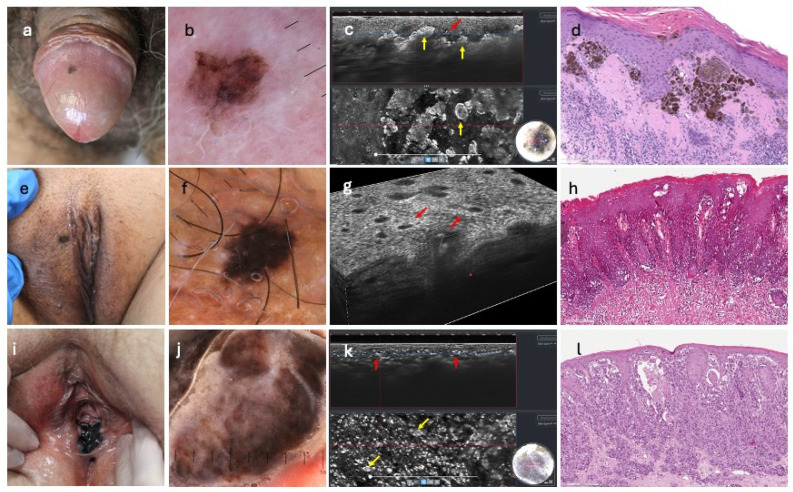
Clinical, dermoscopy, bi-dimensional, 3D LC-OCT, and histopathology of atypical genital nevus, melanoma in situ, and invasive melanoma: (**a**) atypical genital nevus on the glans; (**b**) showing regression and eccentric hyperpigmented areas; (**c**) bi-dimensional LC-OCT revealing bright dense and sparse nests at the junction (yellow arrows), and focal bright pagetoid cells in epidermis (red arrow); (**d**) as observed in histopathology (HE, original magnification × 150); (**e**) melanoma in situ on the pubis; (**f**) with dermatoscopic homogenous pattern; (**g**) 3D LC-OCT cube displaying diffuse atypia at the junction (red arrows); (**h**) corresponding to contiguous proliferation of atypical junctional melanocytes (HE, original magnification × 150); (**i**) invasive melanoma; (**j**) with dermoscopic features of brown structureless areas and fibrosis; (**k**) bi-dimensional LC-OCT clearly showing junctional atypia (red arrows) and irregular nests (yellow arrows); (**l**) relating to dermal proliferation of atypical melanocytes arranged in nests and single lentiginous cells disrupting the normal architecture of the junction (HE, original magnification × 150).

Nevi were mainly defined by a regular epidermis (16/21, 76%), a continuous undulated DEJ (18/21, 86%), and dense melanocytic nests in the superficial dermis (14/21%). Five melanocytic lesions (24%) showed an epidermal disarray with localized or sparse pagetoid cells. Atypia at the junction were detected in 4/21 (19%) nevi, and irregular dermal nests in 7/21 (33%). Specifically, common genital nevi (15/21) were characterized by dense junctional and dermal nests (10/15, 67%), whereas AGN showed discohesive junctional nests (4/6, 67%) (Figure 1 and Figure 2).

AKs were characterized by large dark spaces with a lobular organization in the visible dermis (9/9, 100%), below an acanthotic epidermis (7/9, 78%).

All cases of melanomas were characterized by an irregular epidermis infiltrated by pagetoid large bright roundish cells (10/10, 100%); atypia was also observed along the junction (10/10, 100%), that appeared as a continuous bright line (flat or undulated). Discohesive melanocytic nests were seen in the invasive melanomas (3/10, 30%) (Figure 2).

### 3.2. LC-OCT Horizontal Mode

Melanoses commonly displayed a regular honeycomb pattern (43/56, 77%) overlying a ringed pattern (36/56, 64%), edged papillae (37/56, 66%), and were homogeneously distributed (44/56, 79%). Architectural epidermal disarray with atypical cells was seen in 3/56 (5%) cases, and a draped pattern in 14/56 (25%) lesions; clusters of plump bright cells were seen in 14/56 (25%) lesions (Figure 1).

Nevi were mostly defined by a regular honeycomb pattern (16/21, 76%) in the epidermis and a ringed pattern at the junction (13/21, 62%). Epidermal alterations and pagetoid cell infiltration were seen in 4/21 (19%) nevi. Melanocytic nests in superficial dermis/chorion were observed in all lesions, showing as dense (14/21, 67%), dense and sparse (3/21, 14%), and discohesive (4/21, 19%) (Figure 2).

All melanomas (10/10, 100%) exhibited diffuse epidermal disarray, with widespread pagetoid infiltration of atypical cells and atypia at the junction. A ringed network was seen in 4 of 10 lesions (40%), a meshwork pattern in 5 of 10 (50%); a nonspecific pattern was seen in the remnant case (10%). Non-edged papillae characterized 6 of 10 melanomas (60%), with discohesive nests in 3 of 10 cases (30%) (Figure 2).

In all cases of AKs we observed large dark spaces extending in the dermis (100%). The epidermis consisted of a broadened honeycomb pattern (9/9, 100%).

### 3.3. Multivariate Analysis

Melanoma was most reliably distinguished from benign lesions by the presence of an irregular epidermis, pagetoid cell spreading in both vertical and horizontal sections, and atypical cells at the dermoepidermal junction (DEJ), each observed in all melanomas and in fewer than one quarter of non-melanomas (*p* < 0.001). Non-homogeneous papillary distribution and papillary atypia were likewise strongly indicative of malignancy (100% vs. approximately 16–22%, *p* < 0.001). In post hoc comparisons, these parameters consistently differentiated melanomas from benign lesions (*p* < 0.008). Furthermore, the presence of discohesive melanocytic nests (30% vs. 4.7%, OR 8.8, *p* = 0.02), keratinocyte disarray (50% vs. 16.3%, OR 5.1, *p* = 0.02), and a DEJ meshwork pattern (50% vs. 8.1%, OR 11.3, *p* = 0.002) further supported the diagnosis of melanoma.

### 3.4. Diagnostic Performance of Dermoscopy and LC-OCT

Sensitivity, specificity, and overall diagnostic accuracy of dermoscopy and LC-OCT for the different PGLs are listed in Table 4.

For each lesion subtype, diagnostic accuracy reflected the ability of LC-OCT or dermoscopy to correctly identify that specific entity when evaluated against all other PGLs in the cohort. LC-OCT achieved superior diagnostic accuracy compared with dermoscopy for melanosis (93.8%, 95% CI 88.1–98.4, vs. 90.6%, 95% CI 84.2–96.2), for compound nevi (100%, 95% CI 100–100, vs. 97.9%, 95% CI 93.6–100), and for AGN (96.9%, 95% CI 91.0–100, vs. 90.6%, 95% CI 83.8–97.4). When evaluating melanoma, LC-OCT correctly identified all cases (sensitivity 100%, 95% CI 69.2–100), whereas dermoscopy reached a sensitivity of 70.0% (95% CI 34.8–93.3). In the assessment of angiokeratomas, both modalities yielded excellent diagnostic accuracy, with LC-OCT achieving 100% (95% CI 100–100) and dermoscopy 98.9% (95% CI 94.2–100).

Diagnostic concordance between LC-OCT and histopathology was 90.6% (87/96; 95% CI 83.7–95.3), as nine benign PGLs (six melanoses and three AGNs) were deemed to be melanoma by LC-OCT evaluators.

ROC analysis demonstrated that LC-OCT achieved an excellent ability to discriminate between benign and malignant PGLs, with an area under the curve (AUC) of 0.9477 (95% CI 0.915–0.980). Dermoscopy showed a lower discriminative performance, with an AUC of 0.7628 (95% CI 0.608–0.918).

## 4. Discussion

The higher mortality of melanoma arising in the genital area is largely due to a delay in the diagnosis or an advanced stage at presentation [20,21]. It is an aggressive tumor with a low overall 5-year survival, as prognosis is strictly related to early recognition and a multidisciplinary approach [11,22]. Physicians should be aware that melanoma and benign PGLs may present overlapping clinical features, and the traditional rule of ABCDEs (asymmetry, border irregularity, color variation, diameter > 6 mm, evolution, or changing lesion) is not so useful as for other body sites [7,9,23]. For instance, melanosis frequently presents as a relatively large macule, often exceeding 7 mm in diameter at the time of clinical observation, and shows color changes and/or increase in size over time in 30% of cases, although the course of this entity is benign [8,23]. Dermoscopy can aid in the differential diagnosis as blue, gray, or white colors combined with structureless areas represent an important clue suggestive of melanoma [7,8]. Whether available, RCM should be considered an adjunctive diagnostic tool for a more complete evaluation of PGLs [24,25,26]. As for the common skin, melanoma in the genital area is mainly defined by large pagetoid cells in the epithelium, numerous atypia at the junction, and loss of normal architecture of dermal/chorion papillae [8,9]. However, the standard wide-probe device requires a flat contact area, making it difficult to image curved or narrow surfaces such as the labia or inner foreskin. Hand-held probes aid in solving this technical issue; however, they cannot generate large mosaics [27]. By offering a high resolution and deeper tissue penetration, LC-OCT made it possible to visualize both the epidermis and the underlying DEJ and dermis, correctly identifying density and location of atypical cells [16,28,29,30,31,32].

In this study, LC-OCT consistently had greater diagnostic performance than dermoscopy in the evaluation of benign and malignant PGLs. In addition, an excellent diagnostic concordance between LC-OCT and histopathology (91%) was achieved thanks to the LC-OCT’s in vivo detection of the main pathological criteria for each entity, thus producing a “virtual biopsy”. Indeed, in melanoma, an increased number of intraepidermal melanocytes (pagetoid fashion) and at the DEJ were detected as large bright nucleated cells disrupting the classic architecture of the epidermis and of the junction. Invasive melanoma were additionally characterized by clusters of bright cells, loosely aggregated, forming discohesive nests [33] within the dermis. The morphology of the nests is crucial to making a distinction between nevi and AGN, as nevi are defined by dense nests, while AGN by discohesive nests. However, differential diagnosis between melanoma and AGN remains troublesome also with this imaging technique, since the latter commonly show focal pagetoid spreading and a junctional component composed of large melanocytic nests, with an irregular and variable size and shape. In our series, three AGNs were diagnosed as melanoma for the detection of such architectural irregularities. It should also be noted that the standard histopathological diagnosis of AGN can be difficult due to architectural disarray, the presence of nested growth patterns, and varying degrees of pagetoid spread and cellular atypia [9,33,34].

Classical histopathological features of genital melanosis [35] were clearly identified in LC-OCT, being the elongation of the rete ridges and hyperpigmentation of the basal layer, observed as a bright undulated line in the vertical view, and a ringed or draped pattern in the horizontal view. In this entity, junctional atypia and large bright epidermal cells may represent a diagnostic pitfall, leading to a misdiagnosis of melanoma, as occurred herein in six cases.

Regarding AKs [36], large vascular dermal lacunae were clearly defined with LC-OCT.

The results of this study confirm the clinical relevance of LC-OCT for the evaluation of PGLs, expanding on previous observations regarding the diagnostic challenges in this anatomically and biologically distinct site [19].

Compared with dermoscopy, LC-OCT provided a clearer visualization of epidermal and junctional architecture, allowing for the more reliable identification of atypical melanocytic proliferations and improving diagnostic accuracy across several lesion categories.

The particularly high sensitivity observed for melanoma underscores the potential role of LC-OCT as an adjunctive tool in cases where dermoscopy is inconclusive, especially given the prognostic importance of the early recognition of genital melanoma [1,37].

Using LC-OCT in anatomically restrictive areas (e.g., mucosal areas, interdigital spaces, and ear folds) presents challenges mainly related to probe size, patient positioning, and imaging stability. Also, variability in the interpretation of images among operators may be considered, as 2D images (horizontal and vertical) only provide data from a thin slice. Three-dimensional reconstruction eliminates the potential for the misinterpretation of flat images, providing a volumetric and anatomical understanding of the lesion.

The limited number of melanomas included in this study should also be acknowledged, and larger multicentric cohorts will be required to confirm the reproducibility of these findings.

According to these preliminary results, LC-OCT provides in vivo key clues for different PGLs with a resolution close to histopathology. As distinct morphological findings were identified for each entity, this new device may have a valuable role in the clinical management of PGLs, particularly when lesions are multiple and involve large areas, avoiding unneeded invasive procedures, thus limiting a surgical approach when strictly required. Further prospective studies with larger data are needed to validate these findings.

## 5. Conclusions

In conclusion, LC-OCT emerged as a valuable non-invasive tool for the evaluation of PGLs, offering near histologic visualization of epidermal, junctional and dermal structures.

In our series, LC-OCT achieved high diagnostic accuracy and strong concordance with histopahology, outperforming dermoscopy for several diagnostic categories. These results suggest that LC-OCT may refine the diagnostic pathway of PGLs and reduce unnecessary invasive procedures. Larger prospective studies are warranted to validate these findings and to further develop LC-OCT-based diagnostic algorithms for mucosal lesions.

## Figures and Tables

**Table 1:** Demographics of the sample population.

	Tot.	Melanoses	Nevi	Melanomas	Angiokeratomas
	*n* = 88	*n* = 56	*n* = 21	*n* = 10	*n* = 9
F	65 (74%)	37 (66%)	14 (67%)	10 (100%)	2 (22%)
M	23 (26%)	19 (34%)	7 (33%)	-	7 (78%)
Labia majora		17/37 (46%)	5/14 (36%)	-	-
Labia minora		8/37 (21%)	4/14 (29%)	5/10 (50%)	-
Clitoris		1/37 (3%)	2/14 (14%)	-	-
Pubis		7/37 (19%)	1/14 (7%)	5/10 (50%)	2/9 (22%)
Perianal		4/37 (11%)	2/14 (14%)	-	-
Glans penis		3/19 (16%)	2/7 (29%)	-	-
Prepuce		3/19 (16%)	1/7 (14%)	-	-
Penis		9/19 (47%)	3/7 (43%)	-	1/9 (11%)
Pubis		-	-	-	-
Scrotum		4/19 (21%)	1/7 (14%)	-	6/9 (67%)

N (%) presented in each box.

**Table 2:** LC-OCT vertical-mode criteria.

	Melanoses	Nevi	Angiokeratomas	Melanomas	*p*
	*n* = 56	*n* = 21	*n* = 9	*n* = 10	
Epidermis					
Regular epidermis	43 (77)	16 (76)	2 (22)	0 (0)	<0.001
Irregular epidermis (broadening, dyskeratosis)	10 (18)	5 (24)	7 (78)	10 (100)	<0.001
Pagetoid spread	3 (5)	5 (24)	0 (0)	10 (100)	<0.001
Atypical nests	0(0)	0(0)	0 (0)	2 (20)	<0.001
CEJ and Upper/Mid-Dermis					
Flat	9 (16)	3 (14)	9 (100)	5 (50)	<0.001
Undulated	47 (84)	18 (86)	0 (0)	5 (50)	<0.001
Continuous	56 (100)	21 (100)	9 (100)	7 (70)	<0.001
Atypical cells at DEJ	12 (21)	4 (19)	0 (0)	10 (100)	<0.001
Melanocytic Nests at CEJ and Papillary Dermis					
Dense	0 (0)	14 (67)	0 (0)	0 (0)	<0.001
Dense and sparse	0 (0)	3 (14)	0 (0)	0 (0)	0.01
Discohesive	0 (0)	4 (19)	0 (0)	3 (30)	<0.001
Circular/Ovoidal Dark Spaces	0 (0)	0 (0)	9 (100)	0 (0)	<0.001
Plump Bright Cells	14 (25)	4 (19)	0 (0)	5 (50)	0.08

N (%) presented in each box. CEJ, chorion-epidermal junction.

**Table 3:** LC-OCT horizontal-mode criteria.

	Melanoses	Nevi	Angiokeratomas	Melanomas	*p*
	*n* = 56	*n* = 21	*n* = 9	*n* = 10	
Epidermis					
Honeycomb pattern	43 (77)	16 (76)	9 (100)	5 (50)	0.09
Cobblestone pattern	10 (18)	5 (24)	0 (0)	5 (50)	0.05
Disarray (broadening and atypical keratinocytes)	3 (5)	4 (19)	7 (78)	10 (100)	<0.001
Pagetoid spread of atypical cells	3 (5)	4 (19)	0 (0)	10 (100)	<0.001
CEJ and Upper/Mid-Dermis					
Ringed pattern	36 (64)	13 (62)	0 (0)	4 (40)	0.003
Draped pattern	14 (25)	0 (0)	0 (0)	0 (0)	0.01
Meshwork pattern	4 (7)	3 (14)	0 (0)	5 (50)	0.001
Clod pattern	0 (0)	5 (24)	0 (0)	0 (0)	<0.001
Nonspecific pattern	2 (4)	0 (0)	0 (0)	1 (10)	0.46
Papillae					
Edged	37(66)	7(33)	0 (0)	4 (40)	0.74
Non-edged	17(30)	2(10)	0 (0)	6 (60)	0.02
Mixed	2(4)	2(10)	0 (0)	0 (0)	1
Distribution of Papillae					
Homogeneous	44(79)	9(43)	0 (0)		<0.001
Nonhomogenous	12(21)	2(10)	0 (0)		<0.001
Melanocytic Nests at CEJ and Papillary Dermis					
Dense	0 (0)	14 (67)	0 (0)	0 (0)	<0.001
Dense and sparse	0 (0)	3 (14)	0 (0)	0 (0)	0.01
Discohesive	0 (0)	4 (19)	0 (0)	3 (30)	<0.001
Circular/Ovoidal Dark Spaces	0 (0)	0 (0)	9 (100)	0 (0)	<0.001
Plump Bright Cells	14 (25)	5 (24)	0 (0)	5 (50)	0.10

N (%) presented in each box. CEJ, chorion-epidermal junction.

**Table 4:** Diagnostic performance of LC-OCT and dermoscopy.

		Sensitivity (95% CI)	Specificity (95% CI)	Accuracy (%)
Melanoses	LC-OCT	89.4 (78.5–95.0)	100 (91.2–100.0)	93.8
	Dermoscopy	83.9 (72.2–91.3)	100 (91.2–100.0)	90.6
AGN	LC-OCT	50.0 (18.9–73.3)	100 (95.8–100.0)	96.9
	Dermoscopy	16.7 (6.3–54.7)	95.6 (88.8–98.2)	90.6
Common Nevi	LC-OCT	100 (84.5–100.0)	100 (95.1–100.0)	100
	Dermoscopy	86.7 (65.4–95.0)	100 (95.1–100.0)	97.9
Angiokeratomas	LC-OCT	100 (70.1–100.0)	100 (95.8–100.0)	100
	Dermoscopy	88.9 (56.5–98.0)	100 (95.8–100.0)	98.9
Melanomas	LC-OCT	100 (72.2–100.0)	89.5 (81.3–94.4)	90.6
	Dermoscopy	70.0 (39.7–89.2)	82.6 (73.2–89.1)	81.3

## Data Availability

The original contributions presented in this study are included in the article. Further inquiries can be directed to the corresponding author.

## Abbreviations

LC-OCT	Line-field confocal optical coherence tomography
